# *Beauveria bassiana* Xylanase: Characterization and Wastepaper Deinking Potential of a Novel Glycosyl Hydrolase from an Endophytic Fungal Entomopathogen

**DOI:** 10.3390/jof7080668

**Published:** 2021-08-18

**Authors:** Ayodeji Amobonye, Prashant Bhagwat, Suren Singh, Santhosh Pillai

**Affiliations:** Department of Biotechnology and Food Science, Faculty of Applied Sciences, Durban University of Technology, P.O. Box 1334, Durban 4000, South Africa; dejiamobonye@gmail.com (A.A.); pkbhagwat9988@gmail.com (P.B.); singhs@dut.ac.za (S.S.)

**Keywords:** *Beauveria bassiana*, deinking, entomopathogen, endophyte, enzymes, xylanase, alternative application

## Abstract

*Beauveria bassiana* is an entomopathogenic fungus widely used as a biopesticide for insect control; it has also been shown to exist as an endophyte, promoting plant growth in many instances. This study highlights an alternative potential of the fungus; in the production of an industrially important biocatalyst, xylanase. In this regard, *Beauveria bassiana* SAN01 xylanase was purified to homogeneity and subsequently characterized. The purified xylanase was found to have a specific activity of 324.2 U·mg^−1^ and an estimated molecular mass of ~37 kDa. In addition, it demonstrated optimal activity at pH 6.0 and 45 °C while obeying Michaelis–Menton kinetics towards beechwood xylan with apparent *K_m_*, V_max_ and *k_cat_* of 1.98 mg·mL^−1^, 6.65 μM·min^−1^ and 0.62 s^−1^ respectively. The enzyme activity was strongly inhibited by Ag^2+^ and Fe^3+^ while it was significantly enhanced by Co^2+^ and Mg^2+^. Furthermore, the xylanase was shown to effectively deink wastepaper at an optimal rate of 106.72% through its enzymatic disassociation of the fiber-ink bonds as demonstrated by scanning electron microscopy and infrared spectroscopy. This is the first study to demonstrate the biotechnological application of a homogeneously purified glycosyl hydrolase from *B. bassiana.*

## 1. Introduction

In line with the continuous search for novel enzymes, some focus has since been shifted to entomopathogenic [1] and endophytic fungi [2] as novel sources of industrially important biocatalysts. *B. bassiana*, an endophytic fungal entomopathogen, appears to be a notable candidate in this regard, as the fungus has been shown to produce different enzymes in significant quantities and with distinct biochemical properties [3,4]. In addition, its relative safety for human and animal use, together with its well-characterized genome have made this fungus a potential mining site for industrial enzymes and other important biomolecules [5]. *B. bassiana* though widely perceived as an entomopathogenic fungus has also been found to be endophytic and facultatively saprophytic. As entomopathogens, the life cycle of different *B. bassiana* strains are organized and adapted to their invertebrate hosts, while as endophytes, they maintain a symbiotic relationship with their plant hosts. In this regard, previous attention has been mostly focused on some specific enzymes produced by *B. bassiana* being key factors in the fungus’ entomopathogenicity, viz., the chitinases, lipases and proteases [6,7,8]. However, only recently, different strains of *B. bassiana* have been shown to produce some biomass-degrading enzymes, indicating their less elucidated environmental role as a saprophyte [4].

Hence, different production levels of biomass-degrading enzymes including amylase, beta-glucosidase, endoglucanase, and xylanase have been recorded from the fungus in different studies [3,4,9]. Xylanases (EC 3.2.1.8) are the key enzymes that catalyze the cleavage of the β-1, 4 backbone of the complex plant cell wall polysaccharide xylan, which has been identified as the major hemicellulosic constituent found in soft and hardwood, as well as the second most abundant renewable polysaccharide after cellulose. Xylan is a complex linear polymer made up of repeating xylopyranosyl groups substituted at various positions with different acidic and/or sugar compounds [10]. Thus, the complete and efficient enzymatic hydrolysis of this complex polymer demands an array of enzymes with diverse specificity and modes of action. In this regard, the depolymerization action of xylanases on xylan, together with many other accessory enzymes such as acetylxylan esterases, arabinofuranosidase, glucuronidases and more importantly xylosidase results in the change of the polymeric substance into xylooligosaccharides and xylose [11]. Xylanases have since been found useful in biopulping, biobleaching, functional food production, and nutritional improvements of lignocellulosic feeds [10]. Currently, the normal papermaking process demands the use of various chemicals, which has resulted in various environmental challenges. Hence, the paper and pulp industry is paying attention to novel biotechnology methods to replace a portion of their many chemical-based processes. Xylanases are particularly important in pulp biobleaching as better alternatives to the use of these toxic chlorinated compounds. 

However, enzymes need to fulfil several desirable characteristics for their effective and efficient applications in different industrial processes. These requirements which revolve around their robustness under industrial operations may include stability at specific pH or over a wide range of pHs, thermostability, high specific activity, strong resistance to different metals, solvents and other chemical additives. Other specifications may also include their cost-effectiveness, eco-friendliness, ease of use and recyclability. Hence, the need arises to evaluate the biochemical characteristics of these biological catalysts in order to optimize their performance to suit various applications. Many studies have evaluated the properties of various enzymes in their crude form; however, purification of enzymes has been noted to be essential for detailed biochemical, biophysical and molecular characterization. Although the ultimate degree of purity of a specific protein or enzyme is a function of its end use, purification to homogeneity is highly desirable, despite being highly expensive, laborious and time-consuming [12]. It also helps determine the enzyme’s structure at the primary, secondary, tertiary and quaternary levels. Although successful attempts have been made at the purification of chitinases [13], lipases [6] and proteases [4] from *B. bassiana*, there are currently no reports on the purification of any plant biomass-degrading enzyme from the fungus. Hence, to the best of our knowledge, this is the first report on the purification and characterization of xylanase, or any carbohydrase, from *B. bassiana* SAN01. Furthermore, the study also evaluated the applicability of the fungus in the deinking of wastepaper through the enzymatic action of its xylanase.

## 2. Methodology

### 2.1. Chemicals and Reagents

The wheat bran used in the study was obtained locally in Durban, South Africa. Beechwood xylan, yeast extract and 3,5-dinitrosalicylic acid (DNS) were purchased from Sigma-Aldrich, South Africa. All other chemicals and reagents used were of analytical grade and were obtained from validated suppliers.

### 2.2. Microorganism

*Beauveriabassiana* SAN01 (Gene Accession Number: MN544934) was obtained from the culture collection of the Department of Biotechnology and Food Science, Durban University of Technology, South Africa. The fungus was grown on potato dextrose agar at 30 °C for 7 days and the inoculum was prepared by suspending the spores in sterile 0.1% Tween 20 to give a final count of 1 × 10^7^ spores mL^−1^.

### 2.3. Enzyme Production

Fermentation was carried out in 250 mL Erlenmeyer flasks containing 100 mL of previously optimized media [14]. The media was autoclaved at 121 °C for 20 min and subsequently inoculated with 1 mL of the spore suspension (1 × 10^7^ spores mL^−1^) and incubated at 150 rpm for 12 days. Subsequently, culture broths were filtered and centrifuged at 10,000× *g* for 15 min at 4 °C. The supernatants thus obtained were used as the crude enzyme for subsequent analyses.

### 2.4. Enzyme Assay

Xylanase activity was measured using 1% beechwood xylan (*w/v*). The reducing sugars released during the reactions were quantified by the DNS method, according to Bailey, et al. [15]. One unit of xylanase activity is defined as the amount of enzyme liberating 1 µmol of xylose from the substrate per min under standard assay conditions (40 °C, 50 mM acetate buffer, pH 5.5).

### 2.5. Protein Estimation

Protein concentration in the culture filtrates was estimated according to a modified Lowry method using bovine serum albumin as the standard [16].

### 2.6. Enzyme Purification

#### 2.6.1. Ammonium Sulphate Precipitation

The crude enzyme was purified by precipitation, according to Guillaume, et al. [17]. Solid ammonium sulphate was added slowly to the crude enzyme with gentle stirring to bring to 30% saturation (fraction I), 60% saturation (fraction II), and 90% saturation (fraction III) sequentially. The precipitates obtained from each saturation were dissolved in a minimal volume of 50 mM sodium acetate buffer (pH 5.5) and dialyzed against the same buffer for 24 h at 4 °C.

#### 2.6.2. Ion-Exchange Chromatography

Ion-exchange chromatography was performed according to Kohli, et al. [18] using an ÄKTA protein purifier system (GE Healthcare Life Sciences, Piscataway, NJ, USA). The precipitated active fraction was dialyzed and loaded to Q Sepharose Fast Flow column (5 mL) previously equilibrated with 20 mM sodium acetate buffer (pH 5.5). A continuous NaCl gradient (0–1 M) was used for elution and 1.5 mL fractions were collected. Fractions showing significant xylanase activity were pooled, subsequently concentrated, and desalted using Amicon Ultra-0.5 Centrifugal Filters, with 3 kDa molecular weight cut-off.

#### 2.6.3. Size Exclusion Chromatography

Concentrated fractions obtained after ion-exchange chromatography were filtered under vacuum through 0.45 μm pore size filters (Millipore) to remove any suspended particles. Size exclusion chromatography was carried out on a Superdex^®^ 200 10/300 GL (GE Healthcare Life Sciences) column, using the ÄKTA purifier. A concentrated sample was injected into the system, while NaCl (0.15 M) in sodium acetate buffer (20 mM, pH 5.5) served as the elution buffer [19]. Fractions (0.2 mL) were collected and the ones with significant xylanase activity were pooled and used for further analysis.

### 2.7. Characterization of Enzymes

#### 2.7.1. pH Optima and pH Stability

To determine the optimum pH of the *B. bassiana* xylanase, enzyme activities were evaluated at different pHs using the following buffer systems: acetate (pH 3–6), phosphate (pH 7), Tris–HCl (pH 8–9) and Glycine–NaOH (pH 10). For the pH stability evaluation, the enzyme was pre-incubated at the pHs mentioned above for 30 to 240 min at 30 min intervals, followed by measuring the residual enzyme activities [20].

#### 2.7.2. Temperature Optima and Thermostability

The activities of the enzyme were investigated by incubating the reaction mixtures at different temperatures. For temperature optima, enzyme activities were determined between 25 and 70 °C at 5 °C intervals. In addition, the thermostability of the enzyme was evaluated by pre-incubating aliquots between 25 and 60 °C for 30 to 180 min, at 30 min intervals and measuring residual activities [21].

#### 2.7.3. Effect of Metal Ions and Salt Concentration on Enzyme Activity

The effects of various metal ions including Ag^2+^ (AgNO_3_), Ba^2+^ (BaCl_2_), Co^2+^ (CoCl_2_), Cu^2+^ (CuSO_4_), Fe^2+^ (FeSO_4_), Fe^3+^ (FeCl_3_), Hg^2+^ (HgCl_2_), Mg^2+^ (MgSO_4_), Na^+^ (NaCl), and Zn^2+^ (ZnSO_4_) were determined at 1 mM and 10 mM concentration by adding them to the enzyme reaction mixture and incubating under standard assay conditions [22].

#### 2.7.4. Effect of Various Additives on Enzyme Activity

The effects of various additives including β-mercaptoethanol (BME), dithiothreitol (DTT), ethylenediamine tetra-acetic acid (EDTA), phenylmethylsulphonyl fluoride (PMSF), sodium dodecyl sulphate (SDS), Tween 20 and Triton X-100 were determined at 1 mM and 10 mM concentration by adding them to the enzyme reaction mixture and incubating under standard assay conditions. 

#### 2.7.5. Effects of Different Solvents on Enzyme Activity

The effects of different organic solvents, including acetone, benzene, butanol, chloroform, ethanol, hexane, isopropanol, methanol and toluene were evaluated. Enzymes were incubated in solutions containing each solvent (10%, *v/v*) at room temperature with constant agitation at 120 rpm for 1 h. Subsequently, aliquots were withdrawn and the relative activities were determined under standard assay conditions [23]. 

### 2.8. Kinetic Study and Substrate Specificity

To evaluate the substrate specificity of *B. bassiana* SAN01 xylanase, different reaction mixtures containing the same amount of enzyme and an equimolar amount (10 µM) of specific substrate were incubated under the standard assay conditions. The substrate included arabinoxylan, beechwood xylan, oat spelt xylan, carboxymethylcellulose (CMC), pectin from citrus peel, and soluble starch. The Michaelis constant (*K_m_*) value of the purified enzyme was estimated using a range of beechwood xylan concentrations between 0.1–20 mg·mL^−1^. The apparent *K_m_* value of the purified enzyme was calculated from the Hanes-Woolf plots relating [S]/v to [S] [24]. Furthermore, the *k_cat_* (enzyme turnover number) and the catalytic efficiency were calculated accordingly [25].

### 2.9. Analysis of Protein Pattern by SDS-PAGE

The molecular mass of the purified *B. bassiana* SAN01 xylanase, was evaluated by SDS–PAGE. The analysis was carried out on a Mini PROTEAN gel electrophoresis unit (Bio-Rad, Hercules, CA, USA) using 12% cross-linked polyacrylamide gels and 5% stacking gel [26]. A wide range (6.5–200 kDa) protein ladder (Thermo Scientific, Waltham, MA, USA) was used as the protein marker. 

### 2.10. Zymogram Analysis of Xylanase

The sample was prepared in the standard SDS-PAGE treatment buffer without boiling and without a reducing agent. Afterwards, a zymogram was obtained using a 10% polyacrylamide gel containing 0.2% beechwood xylan (Sigma Aldrich, St. Louis, MO, USA). After electrophoresis, the gel was immersed for 30 min in 2.5% (*v/v*) Triton-X to renature the enzyme and washed in 50 mM sodium acetate buffer (pH 6.0) before final incubation in the same buffer [27]. After 2 h incubation at 40 °C, the gel was stained with 0.2% Congo red (Sigma-Aldrich, St. Louis, MO, USA) and washed with 1 M NaCl. The gel was finally immersed in 1% acetic acid for better contrast.

### 2.11. Deinking of Wastepaper

#### 2.11.1. Optimization of Deinking Process

The waste printed paper was collected, shredded, diced further into smaller pieces and soaked in warm distilled water for 2 h to remove the adhering surface dust particles. The soaked paper pieces were pulverized using a rotary laboratory blender (Eberbach Corp., Van Buren Charter Township, MI, USA); the resultant pulp slurry was drained using cheesecloth and oven-dried at 45 °C for 4 h. Subsequently, 400 mg dried pulp was incubated in 5 mL sodium acetate buffer (pH 6.0, 50 mM) and subjected to different experimental conditions as summarized in Table 1. For the CCD optimization of the wastepaper deinking, process parameters were selected and their ranges were determined based on preliminary experiments and previous studies [28,29]. These parameters include the enzyme load, incubation time and incubation temperature (Table 1). The design was made of 15 combinations including 5 replicates of the center point; the release of ink and reducing sugars were the expected output. The incubation was done with continuous agitation at 50 rpm while enzyme treatment was terminated by inactivating the enzymes by heating the reaction mixture at 90 °C for 5 min. Heat-denatured xylanase served as the control. After enzymatic treatment, pulp filtrate was separated by centrifugation at 5000× *g* for 5 min and analyzed.

#### 2.11.2. Release of Ink

The deinking of the printed paper was evaluated by recording the absorbance at 596 nm for the release of color (diazo reactive black 5) [30]. All experiments were conducted in triplicate, and their mean values are presented. The reducing sugar released for each sample was also evaluated using the DNS method [15].

#### 2.11.3. Scanning Electron Microscopy

Pulp samples were gradually dehydrated with different gradients of acetone, followed by absolute alcohol, according to Chutani and Sharma [31]. Subsequently, samples were air-dried, coated with a thin gold film and viewed under a scanning electron microscope (SEM) (Zeiss; EVO 18, Jena, Germany) at 20 kV to observe the surface morphology. Electron micrographs were taken at ×500, ×1000, ×2000 and ×5000 magnifications.

#### 2.11.4. FTIR Analysis

The surface functional groups of the enzyme-treated pulp and non-treated control was studied by Fourier transformed infrared (FTIR) spectrometry using the attenuated total reflectance (ATR) measuring cell. The infrared spectrometer (FT-IR CARY 630, Agilent Technologies, Santa Clara, CA, USA) with a range of 650 to 4000 cm^−1^, was used for the samples with an average of 32 scans [32].

### 2.12. Statistical Analysis

All experiments were performed in triplicates, and their average values with standard deviation were presented. One-way analysis of variance (ANOVA) and Student’s *t*-test were used for data analysis. The values *p* < 0.05 were considered significant.

## 3. Results and Discussion

### 3.1. Purification and Characterization of B. bassiana SAN01 Xylanase

#### 3.1.1. Purification of *B. bassiana* SAN01 Xylanase

*B. bassiana* SAN01 xylanase produced under submerged fermentation was purified to homogeneity using a series of purification steps, including ammonium sulphate precipitation, ion-exchange chromatography, ultrafiltration and size exclusion chromatography. The xylanase was precipitated using ammonium sulphate (30–60% saturation) to yield an active pellet with a specific activity of 111.29 and 1.52 purification fold. The salt-precipitated enzyme preparation was then dialyzed and purified further by ion-exchange and size exclusion chromatography using Q Sepharose Fast Flow and Superdex 200 10/300 GL columns, respectively. The specific activity of the xylanase increased with each purification step to the maximum value of 369.39 U·mg^−1^ of protein after size exclusion chromatography. In addition, a 2.8% yield and a purification fold of 4.96 were achieved after the final purification step. The overview of *B. bassiana* SAN01 xylanase purification is summarized in Table 2.

**Figure 1:**
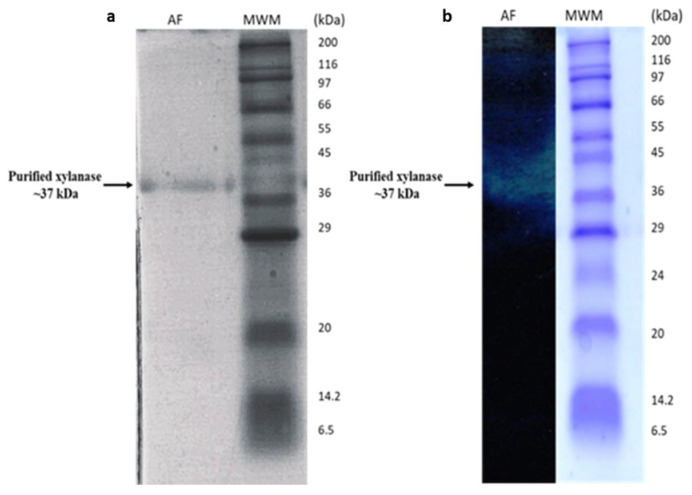
(**a**) *Beauveria bassiana* SAN01 xylanase on 12% SDS-PAGE gel. Lane AF: Active fraction, MWM: Wide range protein marker (ThermoFisher Scientific 6.5–200 kDa) (**b**) Xylanase activity on Native PAGE gel containing 0.5% beechwood xylan, AF: Active fraction, MWM: Wide range protein marker (ThermoFisher Scientific 6.5–200 kDa).

SDS-PAGE analysis of the purified xylanase from *B. bassiana* SAN01 yielded a single band (Figure 1a), which confirmed the homogeneity of the enzyme. The molecular weight of the purified enzyme was estimated to be 36.7 kDa. Xylanases from fungi have been reported to vary from low molecular to high molecular weight proteins. For example, a xylanase of the same size was recently purified from *Aspergillus oryzae* [33], while lower sized xylanases were obtained from *A*. *niger* [34]. Furthermore, the molecular weight obtained in this study is in close agreement with the 37 kDa size of a *B. bassiana* xylanase curated on the UniProt database (UniProt ID: A0A0A2WJL0). The clear zone on the zymogram further confirms the xylanase band on SDS-PAGE and the desired molecular weight of the protein, which corroborates the xylanase activity.

#### 3.1.2. Biochemical Characterization of *B. bassiana* SAN01 Xylanase

**Figure 2:**
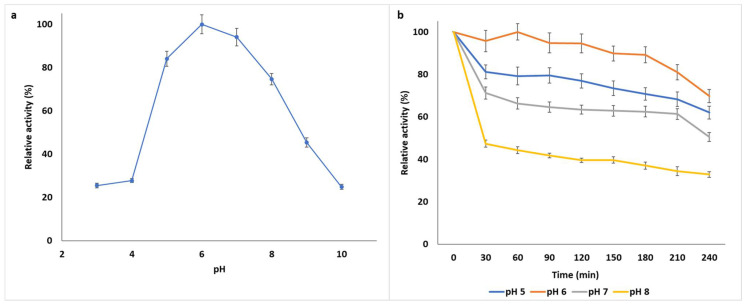
(**a**) Effect of pH on the activity of *Beauveria bassiana* SAN01 xylanase; (**b**) Effect of pH on the stability of *Beauveria bassiana* SAN01 xylanase; each point represents the mean (*n* = 3) ± SD.

Substrate binding and biocatalysis of enzymes are primarily dependent on the charge distribution of the enzyme as well as the substrate molecules, hence enzyme activity is significantly influenced by pH. The optimal pH of the purified *B. bassiana* SAN01 xylanase was observed to be pH 6.0, while it also exhibited significant activities between pH 5 and 8, showing 70 to 100% activity within that pH range (Figure 2a). Beyond this range, the enzyme lost nearly 70% of its activity at pH 4 and above 50% of its initial activity at pH 9. Most fungal xylanases, and enzymes in general, have been noted to be acidophilic [35], although some of these enzymes have also been shown to tolerate alkaline conditions [36]. Previous studies have shown the optimal pH of crude xylanases from other *B. bassiana* strains to be pH 6.0 [4] and pH 6.5 [9]. It was also observed that the enzyme showed greater stability at an acidic range than the alkaline range, as the enzyme retained more than 60% of its activity at pH 5–6 after 2 h incubation (Figure 2b). Furthermore, the enzyme retained 70% of its initial activity at its optimum pH (pH 6.0) after 4 h incubation. The pH stability of xylanolytic enzymes at pH 5.0–7.0 and 5.0–8.0 have been reported from *Penicillium sclerotiorum* [37] and *Rhizophlyctis rosea* [38], respectively. Thus, the enzyme properties suit their application in the food industry, where most of the production processes are in acidic and/or neutral conditions and in the biofuel industry for the hydrolysis of lignocellulosic materials which is also carried out at acidic pHs.

**Figure 3:**
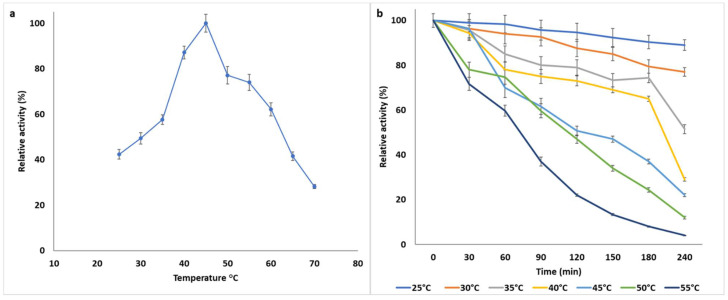
(**a**) Effect of temperature on the activity of *Beauveria bassiana* SAN01 xylanase; (**b**) Effect on temperature on the stability of *Beauveria bassiana* SAN01 xylanase: each point represents the mean (*n* = 3) ± SD.

The optimum temperature of the xylanase was observed to be 45 °C; however, it exhibited remarkable activity between 35 and 60 °C (Figure 3a). Beyond the optimum temperature range, the enzyme activity decreased with increasing temperature, retaining only 30% of the optimum activity at 70 °C. The pH optima of the *B. bassiana* SAN01 xylanase is close to the optimal temperature of 50 °C recorded earlier with crude *Beauveria* sp. MTCC 5184 (Ryali et al., 2020). Other microbial xylanases that are active in the mesophilic range have been described recently. Examples include xylanases from *Penicillium roqueforti* [39] and *Pediococcus acidilactici* [40] with optimal temperatures of 35 and 40 °C, respectively. Furthermore, the purified xylanase was highly stable between 25 and 45 °C (Figure 3b), retaining half of the activity within this range after 120 min. The enzyme was most stable at room temperature, retaining 90% of initial activity after 240 min of incubation; it was observed to retain more than 50% of its initial activity after 120 min at its optimum temperature, 45 °C. At temperatures over 45 °C, the xylanase stability decreased with respect to the incubation time. However, the xylanase from *B. bassiana* SAN01 was more stable than other fungal xylanases reported from *Penicillium sclerotiorum* [37] and *P. chrysogenum* [41], where a larger percentage of their activities were lost within a similar time frame. 

Subsequently, the activating and inhibitory effects of different compounds, including metal compounds, modulators, and organic solvents, were evaluated on *B. bassiana* SAN01 xylanase. The enzyme activity was stimulated by Ba^2+^, Co^2+^ and Mg^2+^ and inhibited by Ag^2+^, Fe^3+^, Hg^2+^, Na^+^ and Cu^2+^, while Fe^2+^ and Zn^2+^ did not alter it significantly (Table 3). It is noteworthy that the enzyme’s activity was increased by Ba^2+^, Mg^2+^ and Co^2+^ (10 mM) to 107.92, 114.97 and 144.49% of its original value. Generally, it can be said that the xylanase was not affected much by the metal ions, as it retained >70% of its enzymatic activity in the presence of most of the ions investigated, at both 1 mM and 10 mM concentrations. The positive effects of Co^2+^ and Mg^2+^ on the activity of xylanase from *Aspergillus ochraceus* [42] and *Halomonas meridiana* [43], respectively, have been reported. Similarly, previous research has also highlighted the inhibitory effects of Ag^2+^ and Na^2+^ on xylanases from *Paenibacillus* sp. [44] and *Thermomyces lanuginosus* [45], respectively. Metal ions have been known to influence the activity of enzymes through various means. The metallic ions accept or donate electrons, activating electrophiles or nucleophiles in the process. They can also act as electrophiles or mask the influence of nucleophiles, thus preventing unwanted side reactions [46]. Besides, they may facilitate enzyme-substrate binding through the co-ordinate bond formation and holding the reacting groups in the optimum orientation. They may also stabilize the catalytically active conformation of enzymes.

Additives such as EDTA, Triton X, SDS, among others, have been shown to have varying effects on different hydrolytic enzymes with accompanying effects during enzyme production or industrial application. The effects of some important chemical additives/reagents on the activity of *B. bassiana* SAN01 xylanase are shown in Table 4. β-mercaptoethanol (BME), dithiothreitol (DTT), and ethylenediaminetetraacetic (EDTA) were observed to significantly enhance the activity of the enzyme. The xylanase activity was increased by 49.33 and 21.14% in the presence of 10 mM BME and DTT, respectively, while the highest increase in activity (~90%) was observed with 1 mM EDTA. Previous studies have shown BME and DTT’s stimulatory effects on xylanase activity [47,48] The stability of xylanase in the presence of BME suggests the absence of any major disulphide bond in the xylanase as BME is a disulphide-bond reducing agent. While the non-inhibitory effect of EDTA on the xylanase activity substantiates the probable absence of metallic prosthetic groups in this enzyme. 

The surfactants evaluated in this study, i.e., SDS, Triton X-100 and Tween 20 exhibited varying inhibitory effects on the enzyme (Table 4). Different xylanases have been noted to be inhibited by these surfactants in recent investigations [48]. It was suggested that the negative effects of SDS on xylanase activity are due to the interference of the detergent in the enzymes’ hydrophobic regions as well as the tertiary structure, leading to its denaturation [33]. However, it was noticed that Tween 20 inhibited *B. bassiana* SAN01 xylanase only at a higher concentration of 10 mM (Table 4). On the other hand, phenylmethylsufonyl fluoride (PMSF) at both lower and higher concentrations reduced the enzyme activity by around 50%. PMSF specifically binds to the serine residues in proteins, and it is a well-known inhibitor of serine/cysteine peptidases, hence, the PMSF inhibition observed in this study may indicate the presence of active site serine residues in the xylanase. The inhibition of fungal xylanases by PMSF was also highlighted by Terrone, Freitas, Terrasan, Almeida and Carmona [41]. 

The effect of various alcoholic and non-alcoholic solvents on *B. bassiana* SAN01 xylanase was also studied. The activity of the enzyme was enhanced in the presence of isopropanol (120%), hexane (120%) and ethanol (110%). In contrast, the enzyme was inhibited in the presence of acetone, butanol, benzene, methanol and toluene (Table 5). Similar observations for xylanase stimulation in the presence of isopropanol, ethanol [49], and hexane [50] were recorded previously. Similarly, xylanases from various sources have also been recently demonstrated to be susceptible to the inhibition by acetone, butanol, benzene, methanol and toluene [48]. In this study, the solvents that significantly enhanced the xylanase activity (ethanol and isopropanol) were more polar than the inhibitory solvents such as benzene and toluene. The relationship between the polarity of solvents and enzyme activity has been highlighted previously [51]. It has also been noted that increased or reduced structural flexibility of enzymes is primarily responsible for the stimulation or inhibition of enzymatic activities, respectively, in organic solvents [52]. Hence, it is posited that the xylanase from *B. bassiana* SAN01 may contain a high proportion of random coils, thus increasing the structural flexibility, which might be responsible for its significant stability in some organic solvents. Biocatalytic reactions in the organic phase offer several advantages such as increased solubility of hydrophobic substrate facilitating effective reactions, lower microbial contamination and recyclability. Hence, the results from this study provide essential data for the applicability of *B. bassiana* SAN01 xylanase in industrial reactions involving organic solvents. Particularly, its significant ethanol tolerance could endear its use in simultaneous saccharification and bioethanol fermentation as well as in the clarification of alcoholic beverages.

#### 3.1.3. Kinetic Analysis of *B. bassiana* SAN01 Xylanase

The kinetic properties of *B. bassiana* SAN01 xylanase were determined using a Lineweaver–Burk plot and the enzyme was observed to obey Michaelis-Menten kinetics. The enzyme was found to have a *K_m_* value of 1.98 mg·mL^−1^ and V_max_ of 6.65 μM·min^−1^. This observed K_m_ is lower when compared to some other fungal xylanases such as xylanase from *Aspergillus niger* [53] and *A. niveus* [54] with *K_m_* values of 2.89 and 18.5. The lower *K_m_* of *B. bassiana* SAN01 xylanase indicates its higher affinity for its substrate, thus the enzyme activity will not be significantly impaired at low substrate concentrations. Furthermore, the catalytic rate constant (*k_ca_*_t_) and the catalytic efficiency (*k_ca_*_t_/*K_m_*) of the enzyme under study were 0.62 s^−1^ and 0.3131 mL·s^−1^·mg^−1^, respectively. The *k_ca_*_t_ represents the amount of bound substrate molecules converted to the desired product per unit time, while *k_ca_*_t_/*K_m_* measures the catalytic efficiency of enzymes for a specific product. 

#### 3.1.4. Substrate Specificity of *B. bassiana* SAN01 Xylanase

The substrate specificity of purified xylanase from *B. bassiana* SAN01 was evaluated against various polymeric substrates. It was observed that the xylanase had high specificity towards xylan-containing substrates (Table 6). *B. bassiana* SAN01 xylanase exhibited substrate specificity in the order: beechwood xylan > oats pelt xylan ˃ arabinoxylan > CMC. Furthermore, the purified enzyme showed no detectable activity towards avicel, pectin and starch, under the same reaction conditions indicating that the xylanase is highly specific for the hydrolysis of hemicellulosic substrates. Similar results were observed in xylanase from *Trichoderma* sp. [55]. Furthermore, it was demonstrated that the enzyme could also act partially on cellulose. 

### 3.2. Deinking of Printed Paper by B. bassiana SAN01 Xylanase

#### 3.2.1. Optimization of Deinking Process

The three selected conditions that affected enzymatic deinking, viz., incubation temperature, time and enzyme load, were optimized to enhance the process. The rate of deinking was observed to vary markedly with the conditions tested in the ranges of 28.27–100% (Table 7), thus indicating the efficacy of *B. bassiana* SAN01 xylanase in removing the ink. The highest clarification efficacy recorded in this study was at 36.47 °C 573.23 U·mL^−1^ enzyme load and 16.25 h incubation. In contrast, the lowest clarification efficacy was observed at the same temperature and enzyme dosage but at a lower incubation time of 7.76 h. The deinking efficiencies of xylanases have been shown in many studies. Xylanases, alone and in combination with other enzymes such as cellulases and laccases, have been used for wastepaper deinking [56,57]. Furthermore, the enzyme has also been coupled with other treatment methods, such as ozone treatment, for deinking both on a laboratory and industrial scale.

It is believed that the enzymes dislodge chromophores from the paper fibers by modifying the ink-fiber surface properties through increased relative hydrophobicity. Furthermore, hemicellulose hydrolysis on the fiber-ink interphase also contributes significantly to the detachment of the chromophores [57]. The efficient removal of these ink compounds from wastepaper will facilitate their recyclability, thus preserving our fragile natural resources by reducing the production of virgin papers from trees and tackling waste generation simultaneously. In addition, a significant amount of reducing sugars was released during the deinking process, ranging between 140.28 and 212.56 mg^−1^. Earlier studies have also highlighted the simultaneous release of reducing sugars and ink chromophores during enzymatic deinking of paper [56]. This could be attributed to the hydrolytic action of the enzyme on the hemicellulosic components of the paper fiber. The release of reducing sugar further emphasizes the valorization of wastepaper in the recycling process as the sugars released could be useful in other downstream industrial processes. Biofuel production using reducing sugars from wastepaper has been a promising approach towards sustainable energy; however, this potential is impeded by the hazardous ink present. Earlier studies have demonstrated the enzymatic saccharification of paper pulp subsequent to bioethanol production [58].

**Figure 4:**
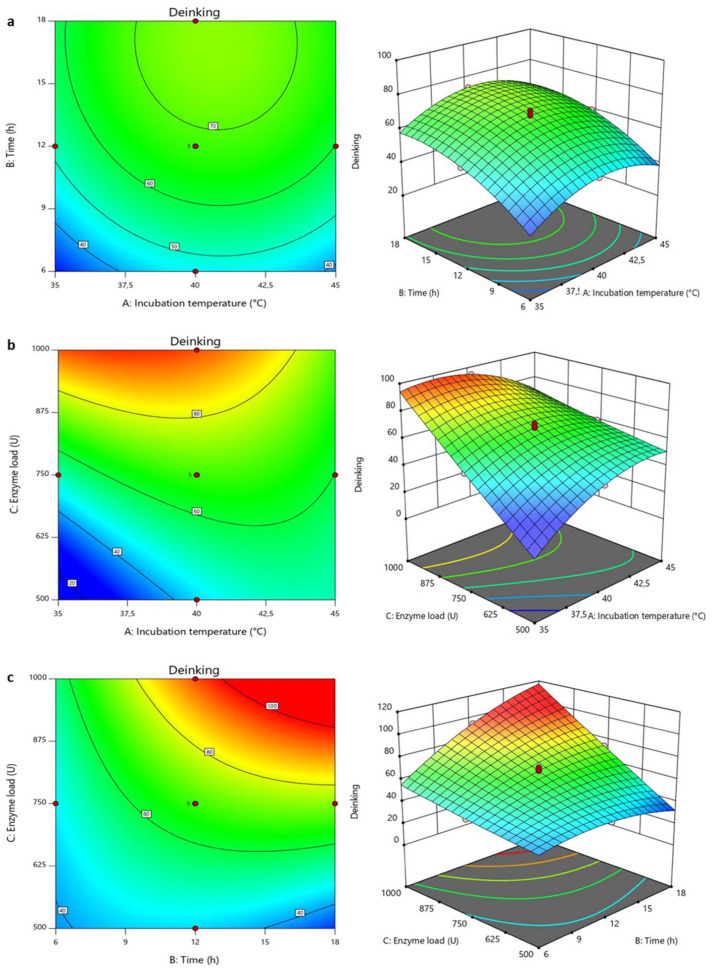
Response surface plots showing the interactions between the variables for *Beauveria bassiana* SAN01 xylanase-assisted wastepaper deinking: (**a**) contour and 3D plots of enzyme load—incubation temperature interaction; (**b**) contour and 3D plots of enzyme load—incubation time interaction; (**c**) contour and 3D plots of incubation time—incubation temperature interaction.

Statistical analysis of the model was performed with the F-test for analysis of variance (ANOVA) as shown in Table S1 (Supplementary Material). The model was confirmed to be valid from the observed F -value (123.02) and *p*-values. The significant effect of all the model terms (A, B, C, AB, AC, BC A^2^, B^2^ and C^2^) were also deduced from their respective *p*-values which were all less than 0.1. The model showed a high *R^2^* value of 0.9955, indicating that the model can account for approximately 100% of the total variation and represent significant relationships among the chosen variables. The effect of the variable changes on the responses could also be observed on the contour and surface plots (Figure 4a–c), showing the effects of temperature, time, and enzyme dose on ink removal from the printed paper. An increase in deinking was recorded with increasing temperature and incubation time until the maximum levels of ~100% were attained at ~40 °C and 17 h. The effects of temperature were observed to be negative beyond the optimum temperature, while time had no significant effects after 17 h. The effects of temperature and reaction time on the enzymatic removal of ink from wastepaper have been highlighted previously [31]. The enzyme load and incubation temperature showed that with an increase in enzyme load, a simultaneous increase in the deinking was observed; however, the efficiency of the enzyme-assisted deinking began to reduce above 40 °C. For every unit increase in temperature, multiplied the effect of enzyme load on the deinking process. As expected, the plots highlighting the interaction between enzyme load and time showed an almost linear relationship between the two factors. The observed response increased with increasing enzyme load and reaction time, even beyond the optimum values.

#### 3.2.2. Validation of the Experimental Model

A validation experiment was carried out to establish the validity of the statistical experimental strategies. The experimental conditions were 40.17 °C incubation temperature, 17.7 h incubation time and an enzyme load of 983.6 U·mL^−1^. The observed value of deinking, 106.72%, was close to that estimated by the RSM model, 113.19%. This demonstrates that the RSM with a central composite design analysis is efficient in optimizing xylanase-assisted deinking of printed paper.

#### 3.2.3. Scanning Electron Microscopy

**Figure 5:**
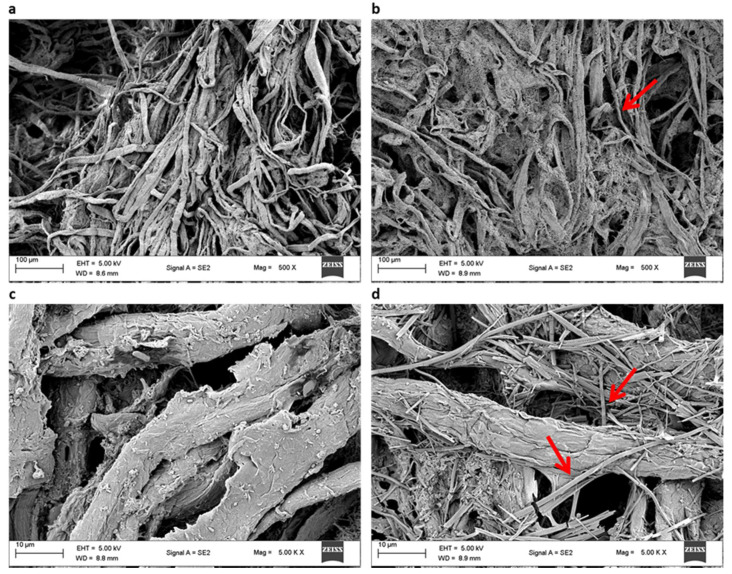
Scanning electron micrographs of (**a**) untreated pulp (500×), (**b**) *Beauveria bassiana* SAN01 xylanase treated (500×), (**c**) untreated pulp (5000×) and (**d**) *Beauveria bassiana* SAN01 xylanase treated pulp (5000×). Arrows indicate fibrillation and perforation on the fiber surface.

The morphological modification of the xylanase-treated paper pulp and untreated control were observed under the scanning electron microscope. The images (Figure 5) confirmed that the xylanase from *B. bassiana* SAN01 significantly modified the structure of pulp fibers. Compared to the control sample, more rugged surfaces, cavities, cracks and disorganized external fibers were observed in the xylanase treated pulp highlighting the alteration of the pulp fibrils at a molecular level resulting from the enzymatic attack. The observed extensive damage and greater porosity signify that *B. bassiana* SAN01 xylanase can degrade the xylan matrix that holds the pulp cellulose microfibrils, leading to surface ink detachment. Similar morphological changes at the ultrastructure level have been observed in enzyme-treated pulps [59,60].

#### 3.2.4. FTIR Analysis

**Figure 6:**
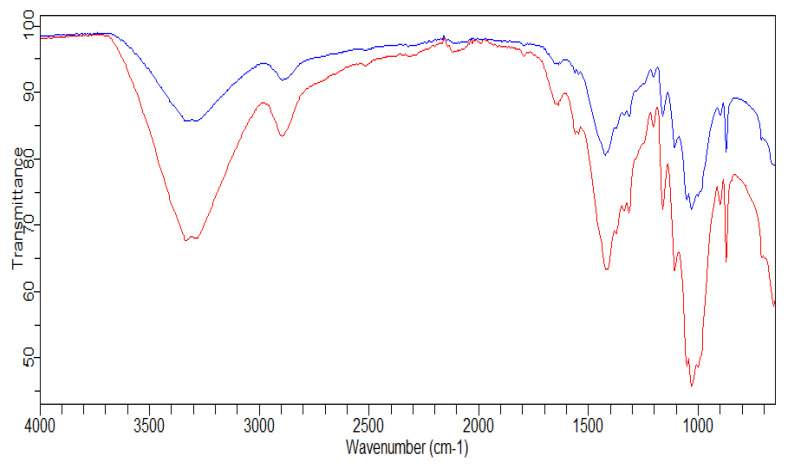
FT-IR spectrum showing the changes in the paper pulp during enzymatic treatment in the range of 4000–600 cm^−1^. *Beauveria bassiana* SAN01 treated pulp **^____^**, Control pulp **^____^**.

The alteration in the structure of xylanase deinked paper samples was characterized using FTIR and compared with the untreated controls (Figure 6). The distinct peak between 3600 and 3200 cm^−1^, which was present in all the paper samples, is ascribed to the hydroxyl groups of cellulose as well as hemicellulose (at 1058, 1315, 898–895 cm^−1^) and lignin content (at 1510–1508, 1605 and 1652 cm^−1^). An earlier study has also shown higher peak intensity at the same wavenumbers in the control pulp in comparison to the treated pulp [61]. The peak at 1434 cm^−1^ is believed to indicate the CH_2_OH group in ink and is observed to be less intense in the test samples. Furthermore, the peaks at 2851 and 2920 cm^−1^ represent the oil content of the ink and they were also of less intensity in the treated samples [58]. These spectra confirm the release of chromophores, hydrophobic compounds and phenolics from printed paper fibers, subsequent to the hydrolytic action of the xylanase. FTIR spectra obtained in this enzyme-assisted deinking are similar to other spectra observed in earlier studies [61,62].

## 4. Conclusions

The biochemical characteristics of *B. bassiana* SAN01 xylanase revealed it to be active and stable over a wide range of pH, from acidic to slightly alkaline. Furthermore, the enzyme was also shown to be moderately thermophilic with its optimum temperature at 45 °C. The purified *B. bassiana* SAN01 xylanase was stable in the presence of various metal compounds, organic solvents and other additives, making it a possible candidate as a biocatalyst in various bioprocesses. Finally, the xylanase was shown to efficiently deink wastepaper with an accompanying release of reducing sugars. Hence, in addition to the established safety of the fungus and its products, findings from this study further underscore the applicability of the fungus in many industrial processes. These findings are expected to stimulate the exploration of *B. bassiana* for industrial enzyme production and other bio-based products along with its well-established use as a biocontrol agent.

## Figures and Tables

**Table 1:** Coded and uncoded variables of the response surface design for *Beauveria bassiana* SAN01 xylanase-assisted paper deinking.

Independent Variables	Units	Levels				
		−1.68	−1	0	+1	+1.68
Temperature (A)	°C	35	36.47	40	43.54	45
Time (B)	h	6	7.76	12	16.25	18
Enzyme load (C)	U·mL^−1^	500	573.23	750	926.78	1000

**Table 2:** Purification table of *Beauveria bassiana* SAN01 xylanase.

Purification Step	Total Activity (U) ^b^	Total Protein (mg) ^c^	Specific Activity (U·mg^−1^)	Yield (%)	Purification Fold
Crude enzyme ^a^	106,100	1452	73.07	100	1
Ammonium sulphate precipitation ^d^	31272	281	111.29	29.47	1.52
Ion exchangechromatography	5047	28.5	177.1	4.75	2.42
Ultrafiltration	4016	14.7	273.2	3.78	3.74
Size exclusion chromatography	2971.6	8.2	362.39	2.8	4.96

^a^ Obtained from 100 mL of cell-free culture extract of *Beauveria bassiana* SAN01 submerged fermentation. ^b^ Enzyme activity measured as described in the methods section. ^c^ Protein concentration determined by Lowry-Hartree assay using BSA as the standard. ^d^ Enzyme was precipitated using ammonium sulphate (30–60% saturation).

**Table 3:** Effect of different metal ions on *Beauveria bassiana* SAN01 xylanase activity.

Metal Ions	Relative Activity (%) ^a^	
	**1 mM**	**10 mM**
Control	100	100
Ag^2+^	64.31 ± 3.18	30.83 ± 1.44
Ba^2+^	94.27 ± 3.13	107.92 ± 6.51
Co^2+^	137.44 ± 7.93	144.49 ± 7.92
Cu^2+^	99.55 ± 4.71	77.53 ± 3.04
Fe^2+^	92.95 ± 4.19	89.86 ± 5.84
Fe^3+^	61.67 ± 3.02	77.09 ± 2.51
Hg^2+^	92.95 ± 4.49	71.80 ± 3.16
Mg^2+^	111.013 ± 5.16	114.97 ± 5.97
Na^+^	85.02 ± 4.02	76.65 ± 3.82
Zn^2+^	101.76 ± 5.15	95.15 ± 4.18

^a^ Data are shown as mean ± SD (*n* = 3); control had no chemical addition.

**Table 4:** Effect of different additives on *Beauveria bassiana* SAN01 xylanase activity.

Additives	Relative Activity (%) ^a^	
	**1 mM**	**10 mM**
Control	100	100
BME	123.34 ± 6.02	149.33 ± 6.27
DTT	109.69 ± 5.63	121.14 ± 5.74
EDTA	189.42 ± 7.33	125.55 ± 6.61
PMSF	55.67 ± 2.68	50.47 ± 3.06
SDS	65.19 ± 2.79	56.38 ± 2.52
Tween 20	103.08 ± 4.37	88.15 ± 4.26
Triton X-100	90.30 ± 4.37	89.86 ± 3.84

^a^ Data are shown as mean ± SD (*n* = 3); Control had no chemical addition.

**Table 5:** Effect of different organic solvents on *Beauveria bassiana* SAN01 xylanase activity.

Organic Solvent	Relative Activity (%) ^a^
Control *	100
Acetone	82.7 ± 4.06
Benzene	81.39 ± 3.88
Butanol	77.20 ± 3.02
Chloroform	86.97 ± 4.42
Ethanol	110.69 ± 5.74
Hexane	104.65 ± 3.63
Isopropanol	120 ± 6.18
Methanol	67.44 ± 1.86
Toluene	87.44 ± 3.86

^a^ Data are shown as mean ± SD (*n* = 3). * Control had no chemical addition.

**Table 6:** Substrate specificity of *Beauveria bassiana* SAN01 xylanase.

Substrate	Relative Activity
Arabinoxylan	76.28 ± 3.36
Avicel	0
Beechwood xylan	100
CMC	17.12 ± 0.56
Oatspelt xylan	87.7 ± 4.16
Pectin	0
Soluble starch	0

**Table 7:** Central composite design for the optimization of *Beauveria bassiana* SAN01 xylanase-assisted printed paper deinking and the response of the dependent variable.

	Level			Deinking (%)			Reducing Sugar (mg/g)
	Coded (A)	Coded (B)	Coded (C)	Actual	Predicted	Actual	Predicted
1	+1.68	0	0	59.68	59.84	167.70	166.64
2	−1	−1	−1	28.27	28.11	121.50	122.56
3	0	−1.68	0	45.83	45.99	174.17	173.11
4	0	0	0	66.90	68.00	198.67	194.28
5	+1	+1	−1	52.08	51.92	168.62	169.68
6	0	0	−1.68	43.33	43.49	140.28	139.22
7	0	0	0	71.16	68.00	190.45	194.28
8	0	0	0	68.27	68.00	187.67	194.28
9	−1	+1	−1	100.00	99.84	196.74	197.80
10	0	0	0	69.04	68.00	197.74	194.28
11	0	+1.68	0	73.45	73.61	202.00	200.94
12	0	0	0	64.97	68.00	194.77	194.28
13	0	0	+1.68	94.32	94.48	212.56	211.50
14	−1.68	0	0	51.66	51.82	159.78	158.72
15	+1	−1	+1	53.51	53.35	170.47	171.53

## Supplementary Materials

The following are available online at https://www.mdpi.com/article/10.3390/jof7080668/s1. Table S1: ANOVA of quadratic models for xylanase-assisted printed paper deinking.
