# Supplementary material for: Beauveria bassiana Xylanase: Characterization and Wastepaper Deinking Potential of a Novel Glycosyl Hydrolase from an Endophytic Fungal Entomopathogen
Source: J Fungi (Basel). 2021 Aug 18;7(8):668. doi: 10.3390/jof7080668 (PMC8398892; doi:10.3390/jof7080668)
Supplement: Supplementary file 1 [file jof-07-00668-s001.zip › jof-1336800-supplementary.pdf]

Beauveria bassiana xylanase: characterization and wastepaper deinking potential of a novel xylanolytic enzyme from an endophytic fungal entomopathogen

Ayodeji Amobonye[a], Prashant Bhagwat[a], Suren Singh[a], Santhosh Pillai[a]*

[a]Department of Biotechnology and Food Science, Faculty of Applied Sciences, Durban University of Technology, P O Box 1334, Durban, KwaZulu-Natal, 4000, South Africa.

*Corresponding author. E-mail: santhoshk@dut.ac.za

Amobonye Ayodeji: https://orcid.org/0000-0002-3220-5858

Prashant Bhagwat: https://orcid.org/0000-0001-5790-7824

Suren Singh: https://orcid.org/0000-0003-1064-6297

Santhosh Pillai **:** https://orcid.org/0000-0003-3474-1555

Table S1: ANOVA of quadratic models for xylanase-assisted printed paper deinking

Source	Sum of Squares	df	Mean Square	F-value	p-value
**Model**	4823.68	9	535.96	123.02	< 0.0001
A-Incubation temperature	32.16	1	32.16	7.38	0.0419
B-Time	381.43	1	381.43	87.55	0.0002
C-Enzyme load	1299.99	1	1299.99	298.39	< 0.0001
AB	0.1376	1	0.1376	0.0316	0.8659
AC	121.99	1	121.99	28.00	0.0032
BC	144.69	1	144.69	33.21	0.0022
A^2	285.84	1	285.84	65.61	0.0005
B^2	129.81	1	129.81	29.80	0.0028
C^2	1,86	1	1.86	0.4258	0.5429
**Residual**	21.78	5	4.36		
Lack of Fit	0.2758	1	0.2758	0.0513	0.8319

R^2 = 0.9955, Adjusted R^2 = 0.9874, Predicted R^2 = 0.9872, Adequate precision AP= 42.089. df; degree of freedom. *Significant p- values at P ≤ 0.1
